# Supplementary material for: Caveolar communication with xenobiotic-stalled ribosomes compromises gut barrier integrity
Source: Commun Biol. 2020 May 27;3:270. doi: 10.1038/s42003-020-0994-1 (PMC7253476; doi:10.1038/s42003-020-0994-1)
Supplement: Supplementary file 1 — Supplementary Information [file 42003_2020_994_MOESM1_ESM.pdf]

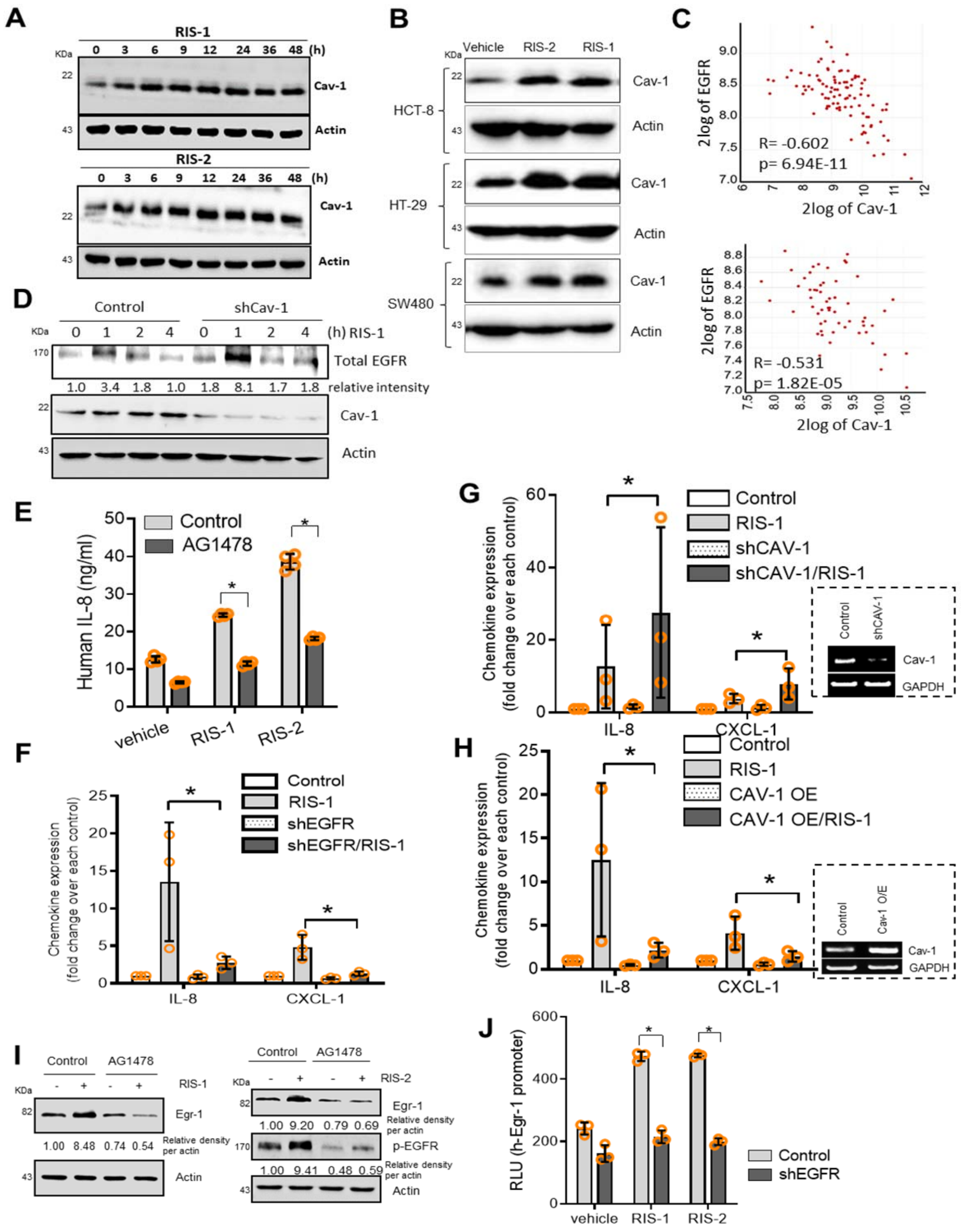

Supplementary Figure 1. (A) HCT-8 cells were treated with vehicle, 50 ng/mL RIS-1, or 500 ng/mL RIS-2 for the indicated time. Cell lysates were subjected to western blot analysis. **(B)** Different intestinal cells were treated with vehicle, 50 ng/mL RIS-1, or 500 ng/mL RIS-2 for 48 h. Cell lysates were subjected to western blot analysis. **(C)** Cav-1 and EGFR expression was compared in patients with IBD based on gene expression arrays (gse75214 [n=95, upper] and gse10616 [n=59, lower]). For two gene correlation coefficient (R) determination, *Pearson*'s correlation analysis was performed. **(D)** HCT-8 cells transfected with control or shCav-1 vector were treated with vehicle or 50 ng/mL RIS-1 for the indicated time. Cell lysates were subjected to western blot analysis. **(E)** HCT-8 cells were pre-exposed to control or 10 µM AG1478 for 2 h and treated with vehicle, 50 ng/mL RIS-1, or 500 ng/mL RIS-2 for 12 h. The IL-8 concentration secreted into the culture media was measured by ELISA. **(F)** HCT-8 cells transfected with control or shEGFR were treated with vehicle or 50 ng/mL RIS-1 for 1 h. IL-8 and CXCL-1 mRNA was measured using real-time RT-PCR. **(G)** HCT-8 cells transfected with control or shCAV-1 were treated with vehicle or 50 ng/mL RIS-1 for 1 h. IL-8 and CXCL-1 mRNA was measured using real-time RT-PCR. Boxes represent relative Cav-1 mRNA levels in HCT-8 cells transfected with control or shCav-1 vector. **(H)** HCT-8 cells transfected with control or Cav-1 overexpression vector were treated with vehicle or 50 ng/mL RIS-1 for 1 h. IL-8 and CXCL-1 mRNA was measured using real-time RT-PCR. Boxes represent relative Cav-1 mRNA levels in HCT-8 cells transfected with control or Cav-1 overexpression vector. (I) HCT-8 cells were pre-exposed to control or 10 µM AG1478 for 2 h and treated with vehicle, 500 ng/mL RIS-2, or 50 ng/mL RIS-1 for 1 h. Cell lysates were subjected to western blot analysis. (J) Control or shEGFR-expressing HCT-8 cells were transfected with human Egr-1 promoter (-1.26kb)-linked reporter plasmid and treated with vehicle, 50 ng/mL RIS-1, or 500 ng/mL RIS-2 for 9 h. (E- I) Groups with an asterisk are significantly different (p<0.05). Results are representative of three independent experiments.

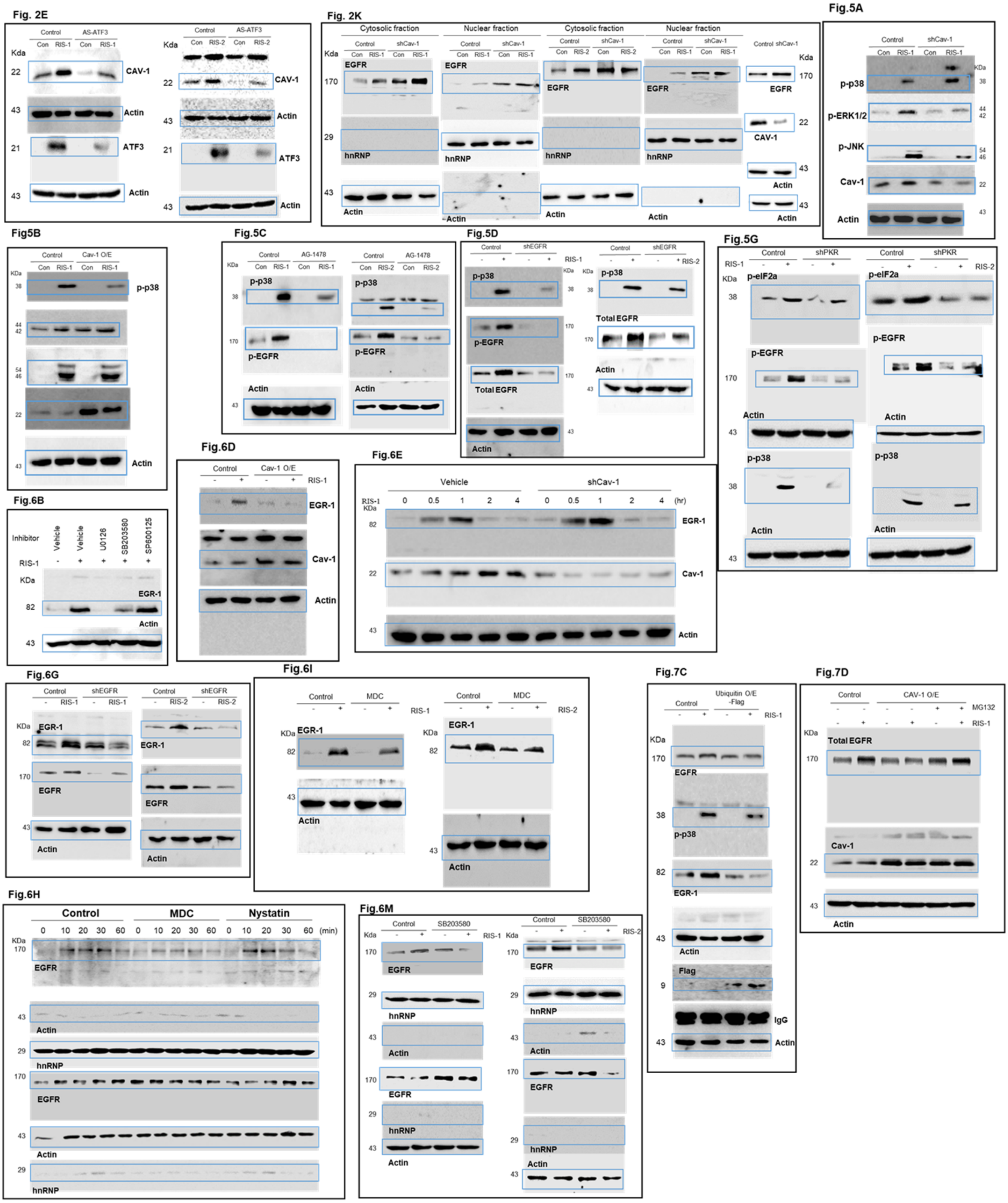

Supplementary Figure 2. Original immunoblots for panels shown in Figures 2, 5, 6, and 7.

Supplementary Table 1. Primers used for PCR

Target genes	sense	Anti-sense
Human IL-8	5'-ATG ACT TCC AAG CTG GCC GTG GCT-3'	5'-TCT CAG CCC TCT TCA AAA ACT TCT C-3'
human CXCL-1	5'-CTG CTC CTG CTC CTG GTA G-3'	5'-TGG ATT TGT CAC TGT TCA GCA-3'
human MCP-1	5’-TCT GTG CCT GCT GCT CAT AG-3’	5’-TGG AAT CCT GAA CCC ACT TC-3’
human Cav-1	5'-TCT CTA CAC CGT TCC CAT CC-3'	5'-CGA AGT AAA TGC CCC AGA TG-3'
human alpha Cav-1	5′-GTC TGG GGG CAA ATA CGT AG-3′	5′- AAG AGG GCA GAC AGC AAG CG -3′
human beta Cav-1	5′-ATG GCA GAC GAG CTG AGC GA-3′	5′-AAG AGG GCA GAC AGC AAG CG-3′
human ATF3	5′-CTC CTG GGT CAC TGG TGT TT-3′ and 5′-AGG CAC TCC GTC TTC TCC TT-3′	5′-AGG CAC TCC GTC TTC TCC TT-3′
human ATF4	5′-GGG CTC ATA CAG ATG CCA CT-3′	5′-CTG ACC ACG TG GAT GAC AC-3′
human GRP78	5′-TGC AGC AGG ACA TCA AGT TC-3′	5′-CGC TGG TCA AAG TCT TCT CC-3′
human CHOP	5′-CTT GGC TGA CTG AGG AGG AG-3′	5′-TCA CCA TTC GGT CAA TCA GA-3′
human Cavin1	5′-ACG AGC AAT ACG GTG AGC AA-3′	5′-CCT CCG ACT CTT TCA GCG AT-3′
human GAPDH	5'-TCA ACG GAT TTG GTC GTA TT-3'	5'-CTG TGG TCA TGA GTC CTT CC-3'
