# Supplementary material for: Caveolar communication with xenobiotic-stalled ribosomes compromises gut barrier integrity
Source: Commun Biol. 2020 May 27;3:270. doi: 10.1038/s42003-020-0994-1 (PMC7253476; doi:10.1038/s42003-020-0994-1)
Supplement: Supplementary file 4 — Reporting Summary [file 42003_2020_994_MOESM4_ESM.pdf]

nature research

Corresponding author(s): YUSEOK MOON

Last updated by author(s): Mar 18, 2020

Reporting Summary

Nature Research wishes to improve the reproducibility of the work that we publish. This form provides structure for consistency and transparency in reporting. For further information on Nature Research policies, see Authors & Referees and the Editorial Policy Checklist.

Statistics

For all statistical analyses, confirm that the following items are present in the figure legend, table legend, main text, or Methods section.

n/a	Confirmed	
☐	☒	The exact sample size (n) for each experimental group/condition, given as a discrete number and unit of measurement
☐	☒	A statement on whether measurements were taken from distinct samples or whether the same sample was measured repeatedly
☐	☒	The statistical test(s) used AND whether they are one- or two-sided<br>*Only common tests should be described solely by name; describe more complex techniques in the Methods section.*
☒	☐	A description of all covariates tested
☐	☒	A description of any assumptions or corrections, such as tests of normality and adjustment for multiple comparisons
☐	☒	A full description of the statistical parameters including central tendency (e.g. means) or other basic estimates (e.g. regression coefficient) AND variation (e.g. standard deviation) or associated estimates of uncertainty (e.g. confidence intervals)
☐	☒	For null hypothesis testing, the test statistic (e.g. F, t, r) with confidence intervals, effect sizes, degrees of freedom and P value noted<br>*Give P values as exact values whenever suitable.*
☒	☐	For Bayesian analysis, information on the choice of priors and Markov chain Monte Carlo settings
☒	☐	For hierarchical and complex designs, identification of the appropriate level for tests and full reporting of outcomes
☐	☒	Estimates of effect sizes (e.g. Cohen's d, Pearson's r), indicating how they were calculated

Our web collection on statistics for biologists contains articles on many of the points above.

Software and code

Policy information about availability of computer code

Data collection	Public datasets (gse117993, gse75214, gse22307, PXD005086) were used.
Data analysis	Analyses were performed using GraphPad Prism v. 5.01 (La Jolla, CA, USA).

For manuscripts utilizing custom algorithms or software that are central to the research but not yet described in published literature, software must be made available to editors/reviewers. We strongly encourage code deposition in a community repository (e.g. GitHub). See the Nature Research guidelines for submitting code & software for further information.

Data

Policy information about availability of data

All manuscripts must include a data availability statement. This statement should provide the following information, where applicable:

- Accession codes, unique identifiers, or web links for publicly available datasets
- A list of figures that have associated raw data
- A description of any restrictions on data availability

All data needed to evaluate the conclusions in the paper are present in the paper. Additional data related to this paper are available from the corresponding author on reasonable request.

Field-specific reporting

Please select the one below that is the best fit for your research. If you are not sure, read the appropriate sections before making your selection.

☒ Life sciences ☐ Behavioural & social sciences ☐ Ecological, evolutionary & environmental sciences

Life sciences study design

All studies must disclose on these points even when the disclosure is negative.

Sample size	For comparative analysis of two groups of data, Student's t test was performed. For comparative analysis of multiple groups, data were subjected to analysis of variance (ANOVA) with Newman-Keuls method as a post hoc ANOVA assessment. For two gene correlation coefficient (R) determination in IBD-based datasets, Pearson’s correlation analysis was performed.
Data exclusions	No data exclusion
Replication	All in vitro evaluations are representative of two or three independent experiments. Details of the number of biological replicates and the assays are given in each figure legends.
Randomization	Random allocation of experimental animals (mice and C. elegans) and cell cultures were applied in all experiments.
Blinding	blinding was employed during data collection.

Reporting for specific materials, systems and methods

We require information from authors about some types of materials, experimental systems and methods used in many studies. Here, indicate whether each material, system or method listed is relevant to your study. If you are not sure if a list item applies to your research, read the appropriate section before selecting a response.

Materials & experimental systems

n/a	Involved in the study	
☐	☒	Antibodies
☐	☒	Eukaryotic cell lines
☒	☐	Palaeontology
☐	☒	Animals and other organisms
☒	☐	Human research participants
☒	☐	Clinical data

Methods

n/a	Involved in the study	
☒	☐	ChIP-seq
☒	☐	Flow cytometry
☒	☐	MRI-based neuroimaging

Antibodies

Antibodies used	The primary antibodies used were rabbit polyclonal anti-β-actin, anti-Cav-1, anti–Egr-1, anti–EGFR, anti–phospho-p38, mouse monoclonal anti-hnRNP and anti-phospho ERK1/2 (Santa Cruz Biotechnology, Santa Cruz, CA, USA), and rabbit polyclonal anti-phospho-EGFR (Epitomics, Burlingame, CA, USA). Anti-flag antibody was purchased from Sigma Aldrich.
Validation	b-Actin 43 kDa https://www.scbt.com/ko/p/beta-actin-antibody-c4?requestFrom=search<br>CAV1 22 kDa https://www.scbt.com/ko/p/caveolin-1-antibody-n-20<br>Egr-1 82 kDa https://www.scbt.com/p/egr-1-antibody-588?requestFrom=search<br>EGFR 170 kDa https://www.scbt.com/p/egfr-antibody-1005?requestFrom=search<br>p-p38 38 kDa https://www.scbt.com/p/p-p38-antibody-thr-180-tyr-182?requestFrom=search<br>hnRNP 29 kDa https://www.scbt.com/p/hnrnp-a1-antibody-4b10?requestFrom=search<br>pERK1/2 44/42 kDa https://www.scbt.com/p/p-erk-antibody-e-4?requestFrom=search<br>p-EGFR 170 kDa https://www.abcam.com/egfr-phospho-y1068-antibody-y38-ab32430.html

Eukaryotic cell lines

Policy information about cell lines

Cell line source(s)	HCT-8, SW480, and HT29 cells were purchased from the American Type Culture Collection (ATCC, Manassas, VA, USA).
Authentication	All cell lines have authentication of the company (American Type Culture Collection (ATCC, Manassas, VA, USA)).
Mycoplasma contamination	All cell lines test negative for mycoplasma contamination.
Commonly misidentified lines (See ICLAC register)	N/A

Animals and other organisms

Policy information about studies involving animals; ARRIVE guidelines recommended for reporting animal research

Laboratory animals	C57BL/6 mice (6 weeks old, 16–18 g on average) were purchased from Jackson Laboratories (Bar Harbor, ME, USA)
Wild animals	N/A
Field-collected samples	Mice were acclimated for 14 days prior to experiments and maintained at 25°C in 45–55% relative humidity under 12 h light/dark cycles. Mice were housed three per cage and provided sufficient food and water in environmentally protected cages comprising a transparent polypropylene body and a stainless steel wire top cover.
Ethics oversight	Animal care and experimental procedures were conducted in accordance with our Institutional Animal Care and Use Committee's guidelines. This animal study was approved by the Pusan National University Institutional Animal Care and Use Committee (PNU-IACUC) (PNU-2015-0786).

Note that full information on the approval of the study protocol must also be provided in the manuscript.
